# A practice-oriented framework for basic life support training of Brazilian school teachers: integrating Law 13,722/2018, andragogy, and the health belief model

**DOI:** 10.3389/fpubh.2025.1616459

**Published:** 2025-10-14

**Authors:** Cyntia Souza Carvalho Castanha, Blanca Elena Guerrero Daboin, Juliana Zangirolami-Raimundo, Luis Fernando Barbosa Tavares, Luiz Carlos de Abreu, Rodrigo Daminello Raimundo

**Affiliations:** ^1^Laboratório de Delineamento de Estudos e de Escrita Científica, Centro Universitário FMABC, Santo André, São Paulo, Brazil; ^2^Centro de Ciências da Saúde, Universidade Federal do Espírito Santos (UFES), Vitória, Espirito Santo, Brazil; ^3^Laboratório de Delineamento de Estudos e de Escrita Científica, Hospital GPACI (Grupo de Pesquisa e Assistência ao Câncer Infantil), Sorocaba, São Paulo, Brazil

**Keywords:** cardiac arrest, basic life support (BLS), CPR training, school teachers, health belief model (HBM), logic model, CIPP model, Brazil Law 13,722

## Abstract

The importance of immediate cardiopulmonary resuscitation (CPR) and proper use of automated external defibrillators in increasing survival rates for cardiac arrest victims is well established. Expanding public access to CPR training and promoting timely emergency response can significantly improve outcomes. Although various global initiatives exist, the inclusion of CPR training in Brazilian school curricula remains limited. The enactment of Law 13,722 in October 2018 marked a major legislative milestone, mandating first aid training for staff in all schools and child recreational facilities as of March 2019. However, implementation challenges persist across Brazil’s diverse and expansive territory. This paper introduces a structured educational framework for Basic Life Support training, incorporating the first three components of the Chain of Survival: recognizing cardiac arrest, activating emergency services, and delivering high-quality CPR. Grounded in Andragogy and the Health Belief Model, the framework emphasizes adult learning principles while enhancing teachers’ knowledge, motivation, self-efficacy, and readiness to act. To support program design and scalability, a Logic Model (resources-to-outcomes map) outlines key implementation steps, while the CIPP model is proposed for monitoring and evaluation. This integrated approach provides a practical, adaptable foundation for implementing school-based emergency preparedness across Brazil, while also creating a scalable pathway that could extend benefits to the broader community through increased CPR readiness.

## 1 Introduction

Prompt and effective cardiopulmonary resuscitation (CPR) and proper utilization of automated external defibrillators (AEDs) are globally recognized interventions for significantly improving survival outcomes in cardiac arrest (CA) victims (1–3). Despite widespread acknowledgement of their life-saving potential, studies show that only about 20% of individuals worldwide are willing or prepared to perform CPR during emergencies (4). Structured CPR training for school teachers has become a common educational practice in high-income countries; however, similar initiatives remain uncommon in low- and middle-income countries due to limitations in resources, trained personnel, and the absence of standardized response curricula within schools (5).

In Brazil, the legislative approval of Law 13,722 in October 2018 marked a significant step toward promoting emergency preparedness within educational settings (6). The law mandates first aid training for staff in schools and child-focused recreational facilities. However, putting this mandate into practice presents challenges, particularly in light of Brazil’s geographic territory and its pronounced socioeconomic and cultural diversity (7). The limited initiatives carried out so far have not demonstrated large-scale impact. For instance, a pilot program introducing CPR training in select schools generated state-level legislation, but its implementation remains localized (8). A recent scoping review further indicates that first aid knowledge among school teachers across both public and private sectors remains generally low (9). These realities underscore that there are no quick fixes: implementing a scalable national program is a long-term effort, especially in resource-limited contexts. This study contributes by creating awareness, providing evidence, and inviting policymakers and stakeholders to act on a law that, despite being enacted in 2019, remains far from fully implemented. Rather than attempting to solve all challenges, the proposal lays the theoretical foundations for scalable and context-sensitive approaches. By linking established behavior change theory with adult learning principles, it provides a roadmap adaptable to Brazil’s regional disparities.

The educational structure is guided by two complementary frameworks, the Health Belief Model (HBM) and Knowles’ Theory of Andragogy. HBM provides a well-established lens for understanding and promoting health behaviors (10, 11). Andragogy ensures that the training is relevant, problem-centered, and builds on teachers’ prior experiences fostering active engagement and improving knowledge retention (12, 13). Together, these frameworks link motivation for behavior change with effective adult learning, providing a comprehensive basis for CPR training in schools.

Two supporting theoretical foundations are also introduced to guide the rollout and assess the program: The Logic Model (LM) is presented as a strategic planning framework to guide the stakeholders involved in systematically aligning program resources, activities, outputs, and expected outcomes (14), and the Context, Input, Process, and Product (CIPP) framework, an evaluative approach, conceptually designed to support the ongoing monitoring, assessment, and continuous improvement of the BLS training once implemented (15).

The authors’ prior experience conducting BLS training with undergraduate medical students (16, 17) provides evidence of the value and effectiveness of structured CPR education. Building on this foundation, the present conceptual approach is designed to support educational institutions, policymakers, and health professionals in planning, implementing, and evaluating BLS training in alignment with Brazilian First Aid Law 13,722/2018. By embedding this legal mandate, the initiative strengthens emergency preparedness in schools and positions teachers as critical first responders. Conceived as an umbrella framework, it offers a flexible structure that can be adapted to Brazil’s heterogeneous school contexts. Operational details, which are context-specific, will be presented in a separate protocol paper, while this manuscript emphasizes the theoretical foundations that enable such adaptation.

## 2 Contextual foundations

### 2.1 School teachers as key lay responders

The “chain of survival” outlines a critical series of actions required to increase the chances of survival in cases of cardiac arrest occurring outside hospital settings (18). Currently, around 80% of cardiac arrests take place in non-hospital environments. The active involvement of lay responders — non-medical individuals can significantly improve the likelihood that CPR is initiated before emergency medical services arrive (19).

The foundation of modern CPR emerged in the early 1960s through the work of Drs. Kouwenhoven, Knickerbocker, and Jude, who introduced closed-chest cardiac massage, alongside Drs. Safar and Elam, who developed mouth-to-mouth ventilation (20). These innovations formed a life-saving technique that could be applied not only by healthcare professionals but also by laypersons in non-clinical settings.

Bystander CPR is defined as: “CPR performed by a person who is not responding as part of an organized emergency response system to a cardiac arrest. Physicians, nurses, and paramedics may be described as performing bystander CPR if they are not part of the emergency response system involved in the victim’s resuscitation. Bystander CPR may be compression-only or include ventilations using rescue breathing or devices” (21).

However, having the technical knowledge and skills to perform CPR or use an AED is only one aspect of effective lay response. Emotional readiness is equally important (19). Research involving interviews with lay responders who intervened during out-of-hospital cardiac arrest (OHCA) events identified five key themes that influence willingness to act: A sense of humanity, perceived competence, feeling a moral obligation, courage, and vulnerability (22).

These factors underline that CPR training programs should not only focus on technical skills, but also consider the importance of psychological readiness in high-stress, life-threatening situations. Although the training proposed here does not explicitly include psychological preparation, this aspect will be monitored as part of the CIPP evaluation model. Insights gathered during implementation may guide the future integration of strategies to strengthen emotional readiness among school teachers.

### 2.2 School teachers as key targets for CPR training

Educational institutions have been recognized as strategic settings for equipping individuals with life-saving skills, such as CPR and the use of AEDs, as recommended by the American Heart Association (AHA). Research has examined both the level of awareness among school teachers regarding CPR and AED use, and the critical role of early defibrillation in improving survival rates following cardiac arrest. These studies reinforce the AHA’s call for integrating CPR and AED training into educational programs AHA (23).

However, many educators report that they do not receive adequate training in these essential competencies, despite their potential role as first responders within the school environment. School teachers are prioritized for CPR training for various reasons, primarily due to their educational qualifications and their consistent presence within school settings where cardiac arrest incidents may occur (24).

Their involvement is emphasized in the ‘Kids Save Lives’ initiative, consequently augmenting the rates of bystander CPR (25). Renowned international organizations, including the AHA (23), the International Liaison Committee on Resuscitation, and the European Parliament (26) advocate for the inclusion of schoolteachers in CPR training, acknowledging their capacity to educate and equip future generations with essential lifesaving competencies.

### 2.3 Andragogy and its contribution to BLS training for school teachers

Malcolm Knowles, a pivotal figure in North American adult education, introduced the concept of andragogy in the late 1960s (12). Initially presented in contrast to pedagogy (12), it is now recognized as a complementary approach that emphasizes the learning process over the simple transmission of content (27). Rooted in the belief that adults are self-directed learners who draw on prior experiences and prefer relevant, problem-solving instruction (28), this framework has remained conceptually stable despite ongoing reinterpretations within adult learning theory (29). Often described as both science and art of helping adults learn (25), it promotes autonomy, experiential learning, and immediate applicability. These principles support the design of realistic, scenario-based sessions that actively engage participants and facilitate the transfer of knowledge to practical, real-world situations.

A key divergence within andragogy lies between two psychological orientations: the humanistic and the behaviorist (30). The humanistic approach emphasizes personal growth, allowing adults to identify their own learning needs. In contrast, the behaviorist perspective stresses structured content, observable outcomes, and reinforcement through repetition or contracts. Knowles (13) explored this tension, contrasting learner-centered facilitation with outcome-focused, behaviorist training.

The application of andragogical principles is particularly effective in the professional development of educators. When applied to the context of CPR training for schoolteachers, andragogy aligns with the goal of not only teaching technical skills but also enabling participants to recognize when and why to act in school-based emergencies. This aligns with adult learners’ preference for practical, problem-solving experiences. Teachers trained using andragogical methods have demonstrated significant improvements in competencies after completing such programs (31, 32).

### 2.4 Legal framework in Brazil

Law 13,722/2018, known as the Lucas Law (33), was driven by the recognition that schools require structured first aid training programs to improve emergency preparedness and response. This need was first articulated in Law Project 9468/2018 (34). The law stipulates that this training must be offered annually and serve both as initial certification and as ongoing recertification for part of the educators and staff in each institution (6).

While this law establishes the mandatory nature of BLS training for teachers and school staff, it does not define a structural training framework such as standardized curricula and minimum course duration. Instead, implementation is delegated to municipal and state authorities, with training commonly delivered through Fire Departments, Municipal Health Departments, Civil Defense units, universities, NGOs, or volunteer organizations (35). In this context, the present conceptual framework provides a common reference model that local authorities and institutions can adapt to their own realities, promoting consistency without disregarding regional differences.

## 3 Challenges in implementing policy in educational settings

In recent years, initiatives in Brazil have promoted BLS training, although with different emphasis. For instance, programs linked to “Kids Save Lives” and university extension projects have introduced CPR education for students and teachers, contributing to awareness and local capacity-building (8, 36). Other studies, such as training of pedagogy students in Paraná, confirm that targeted interventions can increase knowledge and preparedness (37). However, these examples highlight that strategies have been attempted, but scaling and harmonizing them nationally remains a challenge.

Although Law 13,722/2018 mandated first aid training in Brazilian schools, several barriers hinder its effective implementation. These include lack of trained instructors, limited teaching resources, competing demands on teachers’ time, and uneven access to infrastructure across regions (38, 39). Given Brazil’s continental dimensions and geographic heterogeneity, such difficulties are expected: while some urban centers benefit from partnerships with universities or health services, schools in rural and remote areas often lack the minimum resources needed to support regular BLS instruction (40).

Importantly, these barriers are not unique to Brazil. Comparable obstacles limited curriculum time, uneven teacher preparedness, and scarce training resources have likewise been documented in school-based BLS initiatives across Europe, Asia, and North America (41, 42). Similar implementation barriers have also been reported in African countries. In Nigeria, underfunding has restricted the ability of schools to meet education policy mandates (16). In Namibia, shortages of infrastructure and teaching materials, coupled with weak intersectoral coordination, have hindered health policy implementation (39). In Kenya, bureaucratic hurdles have obstructed child-centered policy delivery (40), while in South Africa, teacher overload and overcrowded classrooms have been identified as key obstacles (43).

Conversely, the reported evidence also illustrates best practices that Brazil could adapt. In Europe, the Kids Save Lives initiative shows the impact of embedding CPR training into school curricula with political support from the European Parliament (24). In the United States, state-level mandates requiring CPR instruction in schools demonstrate how legal frameworks can drive large-scale implementation when paired with standardized training materials (44). In parts of Asia, peer-to-peer and community-based training models have effectively expanded reach in resource-constrained settings (45). These experiences suggest transferable strategies for Brazil: systematic curricular integration, refresher training, and scalable peer-to-peer models.

## 4 Theoretical foundations guiding the training design

The proposed training design is anchored in two complementary perspectives: adult learning principles (12, 13) and behavior-change theory (46). In practice, Andragogy shapes how content is delivered while the HBM informs why teachers are motivated to act by addressing perceptions of risk, benefits, and barriers. This integration promotes not only skill acquisition but also the confidence and intrinsic motivation needed to respond effectively in real emergencies. Such alignment reflects contemporary approaches in adult education, which encourage combining theoretical models to address diverse learner needs (27, 47). The six HBM constructs Perceived Susceptibility, Perceived Severity, Perceived Benefits, Perceived Barriers, Cues to Action, and Self-Efficacy — are operationalized in Table 1, which links each construct to specific training activities and content sections (11, 48).

**Table 1:** Application of health belief model constructs to BLS training components.

Section	Activities and materials	Alignment with HBM constructs
1. Introduction and Pre-test	Brief introduction to BLS and The session’s goals. Pre-test questionnaire to assess teachers’ baseline knowledge and motivation.	This section establishes a baseline understanding of participants’ prior knowledge and motivation, helping to tailor the training. Cues to action: Explanation of why BLS is crucial and what they will learn, motivating initial engagement. Perceived susceptibility: Asking about prior experiences and knowledge makes them reflect on their own risk of encountering emergencies.
2. Theoretical Framework	Instructor-led presentation on CA and the importance of BLS. Statistics on global CA deaths, CA data in Brazil, and the reason behind the Law 13,722/2018.	The presentation will highlight the severity of cardiac emergencies and the importance of early intervention. Video testimonials and real-life stories where BLS saved lives will enhance engagement and underscore the significance of BLS skills. Perceived Severity: Data and stories highlight the serious and often fatal consequences of cardiac arrest. Perceived Benefits: Real-life cases of lives saved through BLS show the value of being prepared. Cues to Action: Law 13,722/2018 serves as a legal and social prompt for action.
3. Understanding Risk	Discussion on the risk of medical emergencies in schools and the possibility of anyone experiencing CA, including students and staff.	Emergencies can happen anywhere, even in schools, and they may need to respond in emergencies, reinforcing the sense of susceptibility to these situations. Perceived Severity: Reinforces how serious outcomes can be if no one acts.
4. Role and Impact	Emphasis on the role of being a first responder and the potential for positive outcomes due to early action.	Teachers will discuss the impact they can have during an emergency, reinforcing the importance of their actions (cues to action) and building motivation to engage in CPR confidently (Perceived benefits)
5. Hands-On Practice and Building Confidence	Groups of 4 teachers with 2 mannequins per group (1 adult, 1 child). Practice chest compressions, rescue breaths, AED usage, and choking response techniques. Encouragement to focus on basic CPR skills, even if perfect performance is challenging. Post-test.	Teachers will practice CPR techniques on both adult and child mannequins, addressing differences in approaches for each age group. Instructors will address common concerns or fears about performing CPR to build confidence and reduce perceived barriers. Practicing these skills will build teachers’ confidence in their ability to perform CPR in real scenarios. Emphasis on how even simple actions can save lives will reinforce teachers’ self-efficacy. Perceived Barriers: Addressing fears helps reduce obstacles to action. Cues to Action: Realistic practice serves as a rehearsal for real-life situations. Perceived Benefits: Understanding that even imperfect CPR can increase survival.

The learning frameworks referenced are not rigid or mutually exclusive models. Instead, when combined, they create a robust and responsive approach to adult education. Table 2 illustrates this integration, showing how principles from both Andragogy and the HBM are applied in the design of the BLS training for school teachers.

**Table 2:** Application of andragogical principles complementing the HBM in the design of BLS training for school teachers.

Foundation (andragogy)	Key principle/construct	Application for training design	Relevant training sections
Adult learning	Self-directed learning	Teachers identify personal fears or barriers (e.g., “I’m afraid of hurting someone during CPR”) and propose strategies to overcome them. Pre-tests allow participants to set personal learning goals.	1. Introduction & Pre-test
	Readiness to learn	Use of pre-test results and real-life stories to trigger reflection: “Could this happen in my classroom?”	1. Introduction & Pre-test 2. Theoretical Framework
	Immediate relevance	Training content built around school-specific emergencies (e.g., student choking in cafeteria) to ensure direct applicability.	2. Theoretical Framework 3. Understanding Risk
	Experience-based learning	Invite teachers to share personal experiences of emergencies and connect them with CPR principles.	3. Understanding Risk
	Problem-centered orientation	Simulations framed as problems to solve (“Your colleague collapses during recess — what do you do?”).	4. Role and Impact 5. Hands-on Practice
	Active engagement / experiential learning	Teachers practice on adult/child mannequins in groups, rotate roles (rescuer, observer, feedback-giver), and receive immediate instructor feedback.	5. Hands-on Practice
	Internal motivation	Teachers see tangible evidence of their competence. Passing a post-test reinforces the idea: “I am capable of protecting my students and colleagues in an emergency.” It builds confidence, and strengthens intrinsic motivation to act in real emergencies.	4. Role and Impact 5. Post-test

## 5 Program design and monitoring and evaluation tools

### 5.1 Overview of the training program

**Figure 1:**
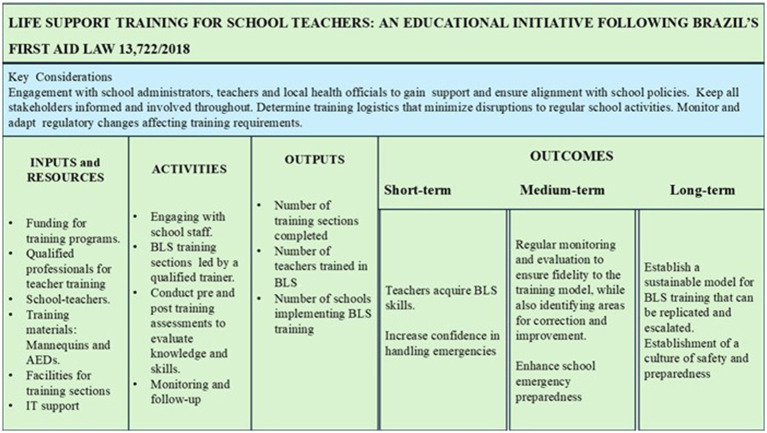
Logic model for BLS training of Brazilian schoolteachers, integrating resources, activities, outputs, and outcomes. The model shows how inputs and training activities translate into short-, medium-, and long-term outcomes, emphasizing stakeholder engagement and regulatory compliance.

The LM depicted in Figure 1 offers a structured, one-page overview of the BLS training program. By linking resources, activities, outputs, and outcomes (14), the LM clarifies the program’s underlying logic and aligns stakeholders during the design phase. Although the LM can also be used for monitoring, in our project it is employed primarily as a communication tool to support planning and development. It also acknowledges the broader context in which the program operates including social, cultural, and political factors, and clarifies how these elements interact with the planned intervention. Importantly, the LM is a flexible instrument that can be readily adapted to both urban and rural Brazilian settings, ensuring that the training remains relevant and applicable across diverse realities. This flexibility arises from the fact that the model is built on stakeholders shared understandings, which anchor the program design in locally defined resources, needs, and priorities (49).

### 5.2 Monitoring and evaluation—the CIPP model

**Figure 2:**
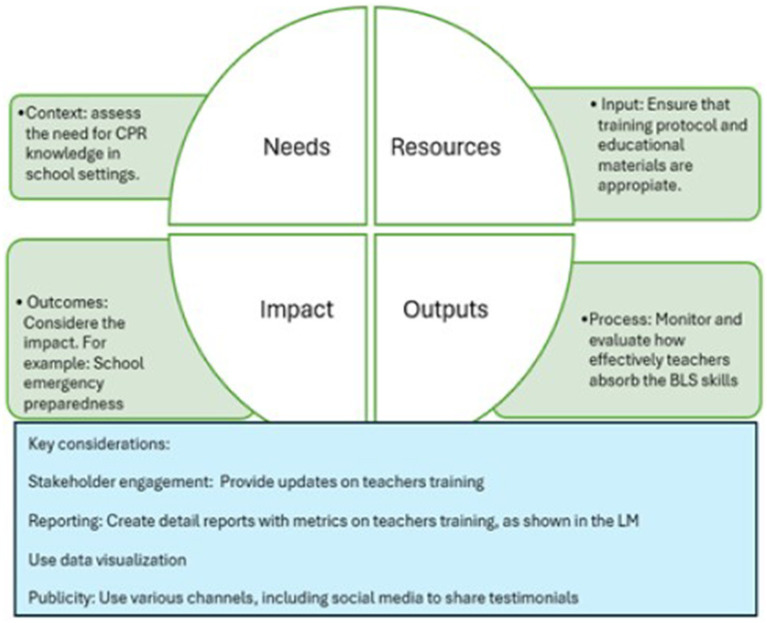
Evaluation tool based on the CIPP model for assessing BLS training implementation in schools. Adapted from Stufflebeam, (2000). Each domain of the CIPP framework is outlined as part of a systematic monitoring and evaluation process.

Measuring, monitoring, and evaluating the effectiveness of an intervention is paramount (50). In alignment with this principle, the CIPP evaluation model (Figure 2) effectively track, monitor, and assess the outcomes of the BLS training program (16, 51). Widely recognized and utilized in both public health and educational fields (48), this approach aligns seamlessly with the LM, ensuring a comprehensive assessment from the initial planning stages, through implementation, to the achievement of desired outcomes (52).

Following the principles outlined by Stufflebeam (53, 54), the CPR training initiative for school teachers (Figure 2) can be contextualized within the CIPP framework as follows: Context Assessment: The objective of this evaluation is to authenticate, comprehend, and document the prevailing circumstances that facilitate the implementation of CPR training (54). Key guiding inquiries encompass: What is the status of emergency preparedness within educational institutions?

What degrees of CPR knowledge or confidence are presently possessed by the school teachers? To what extent are the schools equipped to meet the mandates stipulated by First Aid Law 13,722/2018? Data sources may encompass interviews with stakeholders (including educators and administrators) and reports on school incidents.

Input Evaluation: This evaluation seeks to ascertain that the program inputs are congruent with the intervention as delineated in the LM and are adequately positioned to foster successful implementation Stufflebeam (54). The goal is to verify whether the inputs are both sufficient and appropriate to realize the desired outcomes of the CPR training. Critical components to be observed include the alignment of the curriculum with AHA guidelines and its adaptation to the educational context, the qualifications and readiness of instructors to impart effective training, the availability and suitability of training materials, equipment, and logistical support, as well as compliance with legal and institutional mandates, including adherence to Law 13,722/2018 and any specific school policies.

Process Evaluation: The aim of this phase is to identify real-time opportunities for modification and enhancement, thereby ensuring fidelity to the initial design (54). Are educators attending and actively participating in the sessions? Are trainers delivering the content as intended? Are there obstacles to implementation (e.g., scheduling conflicts, resistance)? Data sources: Observations, post-session evaluations, and attendance records.

Product Evaluation: This phase scrutinizes the outcomes and impact of the program, informing decisions regarding sustainability, modification, scaling, or termination of the program (54). Are educators capable of performing CPR with both confidence and accuracy? Has the overall preparedness of the school improved? Are the acquired knowledge and skills being retained over time?

To move from theory to practice, the CIPP model must be operationalized through clear, measurable indicators and defined follow-up intervals. Table 3 presents how each of the four domains — Context, Input, Process, and Product — has been adapted to the BLS training program. This application ensures that evaluation is not limited to abstract principles but is grounded in concrete measures that capture needs, resources, fidelity of implementation, and both short- and long-term outcomes. By linking the theoretical framework of CIPP with practical indicators, we establish a comprehensive monitoring system that aligns with the requirements of Law 13,722/2018 while also supporting continuous program improvement and sustainability.

**Table 3:** Application of the CIPP evaluation model to the BLS training program.

CIPP domain	Evaluation focus	Example indicators	Timing/follow-up
Context	Identify needs, barriers, assets, and opportunities	% of teachers without prior CPR training Availability of CPR/AED resources (manikins, AED trainers) Number of partnerships established with universities/NGOs/health services	Baseline (before training)
Input	Assess resources, feasibility, and strategies	Teacher: manikin ratio Training cost per teacher Hours of training allocated vs. planned % of schools adopting blended learning vs. traditional	Baseline (planning phase)
Process	Monitor implementation and fidelity	Attendance rate at sessions % of planned activities completed Teacher satisfaction (survey score, 1–5) Number of refresher/practice activities completed	During training & immediately after
Product	Evaluate outcomes, impact, sustainability, transferability	Knowledge gain (pre–post test score improvement) % of teachers performing correct CPR/AED steps in simulation Self-efficacy/confidence scores Skill retention at follow-up Peer-to-peer diffusion: % of teachers involved in training other colleagues or supporting refresher sessions Institutionalization: evidence of annual repetition of training sessions as required by law, and integration of BLS into teacher continuing education programs	Immediate (post), 6 months, 12 months

## 6 Strengths and limitations

This study offers several strengths. First, it integrates Brazil’s legal mandate (Law 13,722/2018) with pedagogical and behavioral theories, ensuring both relevance and theoretical grounding. Second, the use of two complementary models, the LM for program planning and the CIPP framework for evaluation — provides a robust foundation for implementation and monitoring. Third, the framework addresses a critical public health priority by situating BLS training within schools, where teachers can serve as first responders and multipliers of knowledge.

At the same time, some limitations must be acknowledged. As a conceptual paper, this study is intended to clarify constructs, propose a theoretical framework, and lay the foundation for subsequent empirical validation rather than present operational or implementation data (55, 56). Accordingly, the framework has not yet been empirically tested, and its application across Brazil’s diverse educational and infrastructural contexts may face significant challenges. Some of these barriers are already mentioned in Section 3 of this paper. Furthermore, this manuscript does not present cost analyses, logistical considerations, or detailed stakeholder engagement strategies, as these aspects fall outside the scope of a conceptual framework. This does not imply that such factors are unimportant — on the contrary, they are critical and will be addressed in the implementation phase of this umbrella project, which includes a separate protocol paper and pilot evaluation currently under development.

## 7 Conceptual rationale and public health relevance

This framework aligns with Law 13,722/2018 by proposing a structured approach to training school teachers in BLS, equipping them to respond effectively to medical emergencies (57). Implementing CPR training among educators supports scalability and sustainability by leveraging existing school infrastructure and embedding life-saving skills within the education system. Teachers not only act as immediate responders but can also help disseminate knowledge to students over time, fostering a culture of community preparedness. The theoretical foundation of this proposal makes the program both evidence-informed and suitable for long-term evaluation. It represents an initial step in demonstrating the feasibility and public health relevance of BLS training in schools, paving the way for broader institutional adoption.

Its successful implementation, however, will require multi-sectoral collaboration, engaging the education sector, health authorities, and emergency services to ensure alignment of resources, sustainability, and effectiveness.

Looking ahead, future research should focus on pilot testing in diverse school settings, longitudinal evaluation of knowledge and skill retention, and exploration of how BLS training can be formally integrated into teacher certification and continuing professional development programs. In doing so, the model can inform broader health education strategies and help operationalize Law 13,722/2018 across Brazil’s diverse school systems.

## References

[1] Elhussain MO Ahmed FK Mustafa NM Mohammed DO Mahgoub IM Alnaeim NA . The role of automated external defibrillator use in the out-of-hospital cardiac arrest survival rate and outcome: a systematic review. Cureus. (2023) 15. doi: 10.7759/cureus.47721 PMC10676231 38021997

[2] Luo Y Jiang Y. A review regarding the article ‘health inequalities in cardiopulmonary resuscitation and use of automated electrical defibrillators in out-of-hospital cardiac arrest. Curr Probl Cardiol. (2024) 49:102581. doi: 10.1016/j.cpcardiol.2024.102581, PMID: 38653444

[3] Castanha CSC Tavares LFB Leone C Paiva LS Daboin BEG Marques NSF . Basic life support education: the impact of lecture-demonstration in undergraduate students of health sciences. J Hum Growth Dev. (2021) 31:283–90. doi: 10.36311/jhgd.v31.11509

[4] Daud A Nawi AM Aizuddin AN Yahya MF. Factors and barriers on cardiopulmonary resuscitation and automated external defibrillator willingness to use among the community: a 2016–2021 systematic review and data synthesis. Glob Heart. (2023) 18:46. doi: 10.5334/gh.1255, PMID: 37649652 PMC10464530

[5] Schroeder DC Finke S-R Grübl T Jänig CW Böttiger BW. Education of schoolchildren in cardiopulmonary resuscitation–overview of the current literature. Curr Opin Crit Care. (2023) 29:616–20. doi: 10.1097/MCC.0000000000001111, PMID: 37861212

[6] BRASIL. Lei n. 13.722, de 4 de outubro de 2018. Torna obrigatória a capacitação em noções básicas de primeiros socorros de professores e funcionários de estabelecimentos de ensino públicos e privados de educação básica e de estabelecimentos de recreação infantil. Portal da Legislação: Leis Ordinárias. (2018). Available at: https://www.planalto.gov.br/ccivil_03/_ato2015-2018/2018/lei/l13722.htm (Accessed February 15, 2025).

[7] IBGE-Social Indicators. Available online at: https://agenciadenoticias.ibge.gov.br/en/agencia-news.html?editoria=sociais (Accessed February 26, 2025).

[8] Nakagawa NK Silva LM Carvalho-Oliveira R Oliveira KMG Santos FRA Calderaro M . KIDS save lives Brazil: a successful pilot program to implement CPR at primary and high schools in Brazil resulting in a state law for a training CPR week. Resuscitation. (2019) 140:81–3. doi: 10.1016/j.resuscitation.2019.05.009, PMID: 31121207

[9] Silva LC d MA de Melo Alves Silva LC Alves IL dos Santos KVG da Silva TTM Silva Leal KC . First aid teaching for schoolchildren: scoping review. Int. J. Educ. Res. Open. (2023) 5:100305. doi: 10.1016/j.ijedro.2023.100305

[10] Sayre MR Berg RA Cave DM Page RL Potts J White RD. Hands-only (compression-only) cardiopulmonary resuscitation: a call to action for bystander response to adults who experience out-of-hospital sudden cardiac arrest: a science advisory for the public from the American Heart Association emergency cardiovascular care committee. Circulation. (2008) 117:2162–7. doi: 10.1161/CIRCULATIONAHA.107.189380, PMID: 18378619

[11] Green EC Murphy EM Gryboski K. The health belief model In: The Wiley encyclopedia of health psychology. Hoboken, New Jersey: John Wiley & Sons (2020). 211–4.

[12] Knowles M. S. Andragogy, not pedagogy, adult leadership. No volume or page numbers provided. However, a replica may be found on pp. 517–520 of Sopher, MJ (2003). An historical biography of Malcolm S. Knowles: The remaking of an adult educator, 1968.

[13] Knowles MS. The adult learner: A neglected species. Houston, TX: Gulf (1973).

[14] Van Melle E. Using a logic model to assist in the planning, implementation, and evaluation of educational programs. Acad Med. (2016) 91:1464–4. doi: 10.1097/ACM.0000000000001282, PMID: 27332869

[15] Sankaran S Saad N. Evaluating the bachelor of education program based on the context, input, process, and product model. Front Educ. (2022) 7:924374. doi: 10.3389/feduc.2022.924374

[16] Del Águila JG Rebollo EL Pérez RE Gutiérrez ML Del Valle PF Sánchez MG . Teachers’ training of schoolchildren in basic life support. Emergencias. (2019) 31:185–8. 31210451

[17] Tavares LFB Raimundo RD Leone C Castanha CSC Gonçalves de Oliveira A Daboin BEG . Learning assessment from a lecture about fundamentals on basic life support among undergraduate students of health sciences. Healthcare. (2020). doi: 10.3390/healthcare8040379 PMC7711553 33019578

[18] Högstedt A Thuccani M Carlström E Claesson A Bremer A Ravn-Fischer A . Characteristics and motivational factors for joining a lay responder system dispatch to out-of-hospital cardiac arrests. Scand J Trauma Resusc Emerg Med. (2022) 30:22. doi: 10.1186/s13049-022-01009-1, PMID: 35331311 PMC8943963

[19] Nye J. AHA statement on bystander intervention during out-of-hospital cardiac arrest. Medical Bag. (2022) 145:e852-e67. doi: 10.1161/CIR.0000000000001054

[20] Kouwenhoven WB Jude JR Knickerbocker GG. Closed-chest cardiac massage. JAMA. (1960) 173:1064–7. 14411374 10.1001/jama.1960.03020280004002

[21] Perkins GD Jacobs IG Nadkarni VM Berg RA Bhanji F Biarent D . Cardiac arrest and cardiopulmonary resuscitation outcome reports: update of the Utstein resuscitation registry templates for out-of-hospital cardiac arrest: a statement for healthcare professionals from a task force of the international liaison committee on resuscitation (American Heart Association, European resuscitation council, Australian and New Zealand council on resuscitation, Heart and Stroke Foundation of Canada, InterAmerican Heart Foundation, resuscitation Council of Southern Africa). Circulation. (2015) 132:1286–300. doi: 10.1161/CIR.0000000000000144 25391522

[22] Axelsson Å Thorén A Holmberg S Herlitz J. Attitudes of trained Swedish lay rescuers toward CPR performance in an emergency: a survey of 1012 recently trained CPR rescuers. Resuscitation. (2000) 44:27–36. doi: 10.1016/s0300-9572(99)00160-4, PMID: 10699697

[23] American Heart Association. Highlights of the 2015 American Heart Association Guidelines update for CPR and ECC. Available online at: http://www.documentcloud.org/documents/6005325-2015-AHA-Guidelines-Highlights-English.html (Accessed December 2024).

[24] Schroeder DC Semeraro F Greif R Bray J Morley P Parr M . KIDS SAVE LIVES: basic life support education for schoolchildren: a narrative review and scientific statement from the international liaison committee on resuscitation. Circulation. (2023) 147:1854–68. doi: 10.1161/CIR.0000000000001128, PMID: 37194575

[25] Clair RS. Andragogy: past and present potential. New Dir Adult Contin Educ. (2024) 2024:7–13. doi: 10.1002/ace.20546

[26] European Parliament. Parliamentary question | Compulsory first aid instruction in primary schools | E-000410/2020 | European Parliament (2020). Accessed February 15, 2025.

[27] Knowles MS Holton EF III Swanson RA. The adult learner: The definitive classic in adult education and human resource development. New York, NY: Routledge (2014).

[28] Knowles M. S. Holton E. F. III. Swanson R. A. (2005). The adult learner: The definitive classic in adult education and human resource development. San Diego, California: ProQuest EBook Central. Available online at: http://ebookcentral.proquest.com/lib/northeastern-ebooks/detail.action?docID=232125.

[29] Knowles MS. The modern practice of adult education: Andragogy versus pedagogy. New York: Cambridge Book Company (1970).

[30] Hikmah RKA Wahyuni S. Implementation of andragogy approaches in training tourism to develop tourist village In: 6th international conference on education and technology (ICET 2020): Atlantis Press (2020). 312–7.

[31] Irdamurni. •Proceedings article•10.2991/ICSET-17.2017.114. Inclusive education training model based on need assessment and andragogy for elementary school teachers; (2017)

[32] Novitasari D Sugito S. Improving the skill of early childhood education teachers in making lesson plans through an andragogy-based training. Journal of Nonformal Education. (2018). 4:97–106. doi: 10.15294/JNE.V4I1.13578

[33] De Lima Mantovani J Mantovani J d L Mazziero PFE Barbieri MRB Caram ALA Ricci WZ . Avaliação do conhecimento sobre a lei Lucas e sua aplicabilidade: estudo piloto na rede de ensino pública do ensino infantil e fundamental. Arquivos de Ciências da Saúde da UNIPAR. (2023) 27:1946–61. doi: 10.25110/arqsaude.v27i4.2023-022

[34] BRASIL. Projeto de Lei PL 9468/2018. Institui a obrigatoriedade de estabelecimentos de ensino das redes pública e privada voltados à educação infantil e à educação básica e os estabelecimentos de recreação infantil capacitarem profissionais do seu corpo docente ou funcional em noções básicas de primeiros socorros. Available at: https://www.camara.leg.br/proposicoesWeb/prop_mostrarintegra;jsessionid=EBECE0A4E7215071ED74A4C8030A5757.proposicoesWebExterno2?codteor=1639155&filename=PL+9468/2018 (Accessed December 29, 2024).

[35] de Andrade AF Camargo APL de Cácia B Baptista S de Lima Siqueira JE Persuhn RFNS. APLICABILIDADE da lei FEDERAL n° 13.722/2018 (lei LUCAS) e a SEGURANÇA INFANTIL NAS ESCOLAS. Revista Multidisciplinar do Nordeste Mineiro. (2025) 8:1–31. doi: 10.61164/rmnm.v8i1.3867

[36] Tony ACC Carbogim FDC Motta DS Santos KBD Dias AA Paiva ADCPC. Teaching basic life support to schoolchildren: quasi-experimental study. Rev Lat Am Enfermagem. (2020) 28:e3340. doi: 10.1590/1518-8345.4078.3340, PMID: 33027401 PMC7529447

[37] Soares AP Fernandes FECV de Melo RA Torres IA. Impact of BLS training on pedagogy students in Paraná. Res Soc Dev. (2024) 13:e47865. doi: 10.47149/pemo.v7.e13616

[38] Ossa AG. Implementation of education policy in secondary schools in Delta state: challenges and future directions. Tamaddun. (2022) 21:255–63. doi: 10.33096/tamaddun.v21i2.266

[39] Katangolo-Nakashwa N Mfidi FH. Exploring the hurdles of implementing National School Health Policy in Namibian schools: insights from stakeholders. BMC Health Serv Res. (2025) 25:131. doi: 10.1186/s12913-024-12197-0, PMID: 39849533 PMC11759415

[40] Githinji W Ndiangui P. Synthesizing policy and practice: an examination of child-related policy implementation in elementary education within Nyeri County, Kenya. Res Educ Policy Manage. (2024) 6:117–35. doi: 10.46303/repam.2024.26

[41] Rath S Stepaniuk I. The cumbersome burden of translating policy into practice: engaging culturally and linguistically diverse (CLD) families in special education In: Advocating and empowering diverse families of students with disabilities through meaningful engagement. Pennsylvania, USA: IGI Global (2023). 101–16.

[42] Vican D. Inside the school: A comparative review of empirical and policy studies on the role of school leaders in developing schools and teachers In: Educational leadership in policy: challenges and implementation within Europe (2019). 137–52.

[43] Mzeli BA. In Challenges Facing Implementation of New Education Policies in Government Primary Schools: The Case of Fee Free Education Policy in Gairo District. Ingþórsson AH, Alfirević N, Pavičić J, Vican D, editors. Switzerland: Palgrave Macmillan Int J Sci Res. (2023) 12:1928–33. doi: 10.21275/SR231011174635

[44] American Heart Association. CPR in schools. Available online at: https://cpr.heart.org/en/training-programs/community-programs/cpr-in-schools (Accessed December 2024).

[45] Lee MJ Shin TY Lee CH Moon JD Roh SG Kim CW . Steering committee of 2020 Korean guidelines for cardiopulmonary resuscitation and emergency cardiovascular care. 2020 Korean guidelines for cardiopulmonary resuscitation. Part 9. Education and system implementation for enhanced chain of survival. Clin Exp Emerg Med. (2021) 8:S116–24. doi: 10.15441/ceem.21.029, PMID: 34034453 PMC8171173

[46] MohammadiDogahe A Mehrabian F Ashouri A Karimy M Kasmaei P. Designing and evaluating the effectiveness of an educational intervention based on the health belief model (HBM) in promoting self-care behavior among healthcare workers. Jundishapur J Health Sci. (2024) 16. doi: 10.5812/jjhs-146632

[47] Merriam SB Bierema LL. Adult learning: Linking theory and practice. San Francisco: John Wiley & Sons (2013).

[48] Kahnooji Z Mirzaei T Asadpour M Sabzevari S. Effect of educational intervention based on health belief model to promote cardiovascular disease preventive behaviors. Community Health J. (2021) 14:1–12. doi: 10.22123/chj.2021.220985.1449

[49] Frye AW Hemmer PA. Program evaluation models and related theories: AMEE guide no. 67. Med Teach. (2012) 34:e288–99. doi: 10.3109/0142159X.2012.66863 22515309

[50] Sullivan TM Strachan M Timmons BK Rinehart W. Guide to monitoring and evaluating health information products and services. Baltimore, Maryland: Center for Communication Programs, Johns Hopkins Bloomberg School of Public Health (2007).

[51] Kim O-J. A study on the measures for managing the quality of curriculum of early childhood education department in college with the application of CIPP model based on PDCA. J Korea Converg Soc. (2019) 10:215–26. doi: 10.15207/JKCS.2019.10.1.215

[52] Stufflebeam DL. The CIPP model for evaluation In: Evaluation models: Viewpoints on educational and human services evaluation. Dordrecht: Springer Netherlands (2000). 279–317.

[53] Stufflebeam D. L. The relevance of the CIPP evaluation model for educational accountability. Columbus: Educational Accountability. Institution Ohio State Univ., Columbus. Evaluation Center. (1971).

[54] Stufflebeam DL. The CIPP model for evaluation In: Stufflebeam DL Kellaghan T, editors. The international handbook of educational evaluation (chapter 2). Boston: Kluwer Academic Publishers (2003)

[55] Jaakkola E. Designing conceptual articles: four approaches. AMS Rev. (2020) 10:18–26. doi: 10.1007/s13162-020-00161-0

[56] Torraco RJ. Writing integrative literature reviews: guidelines and examples. Hum Resour Dev Rev. (2005) 4:356–67. doi: 10.1177/1534484305278283

[57] Mpotos N Vekeman E Monsieurs K Derese A Valcke M. Knowledge and willingness to teach cardiopulmonary resuscitation: a survey amongst 4273 teachers. Resuscitation. (2013) 84:496–500. doi: 10.1016/j.resuscitation.2013.01.023, PMID: 23376584

